# The Principles of Surgery

**Published:** 1856-07

**Authors:** 


ART. VI.—*The Principles of Surgery.* By JAMES MILLER, F. R. S. E., F. R. C. S. E., Author of a Treatise on the Practice of Surgery, etc., etc. Fourth American, from the Third and Revised English Edition. Philadelphia: BLANCHARD & LEA. 1856.

We are glad to welcome another edition of Miller's Principles of Surgery. Its popularity is a fair index of its merit, and the work is so widely known to American readers that a review is unnecessary.

It is, however, humiliating to the American reviewer to sit down to a table loaded with English, French, and German republications or translations. They are good books, doubtless, and their diffusion among the profession is calculated to benefit American science. But have we no surgeons, no physicians, no microscopists, no pathologists at home? Must we ever go abroad for our scientific literature, as we do for our brandies and silks? Are we but vassals of the scientific aristocracy of the old world?

We know men at our own door capable of better treatises on surgery than any of the last dozen importations. American scientific works—few in number as they are—are ever a matter of pride with us, for we do not hesitate to express the opinion that the profession of this country, as a whole, is superior to that of Europe, and their works have a corresponding value. But where are our writers? Where the spirit of encouragement which they deserve from their countrymen? The few who do write must struggle on under a ruinous competition with imported works, or confine themselves to compends, small books for idle students, or the second-hand glory of patching in a few notes as editors of the "American edition."

We do not say this in depreciation of the enterprize of American publishers, who are constantly throwing out to the profession at cheap, accessible prices, the best works of European intellect. But we would have more culling, fewer republications of books regarded as worthless at home, written by book-wrights without practice, without personal acquaintance with the subjects which they treat—books written as personal advertisements.

If only this class of republications were gotten rid of, the effect would be to open the market to American authors of merit; and only such can succeed under our present system of active, competition and rivalship.

All this has very little to do with Miller's Principles of Surgery, which appears this time without an editor, but we have seized the opportunity to utter a protest against our colonial literary position.

